# Perinatal Trajectories of Maternal Depressive Symptoms in Prospective, Community-Based Cohorts Across 3 Continents

**DOI:** 10.1001/jamanetworkopen.2023.39942

**Published:** 2023-10-26

**Authors:** Michelle Z. L. Kee, Andrea Cremaschi, Maria De Iorio, Helen Chen, Tina Montreuil, Tuong Vi Nguyen, Sylvana M. Côté, Kieran J. O’Donnell, Gerald F. Giesbrecht, Nicole Letourneau, Shiao Yng Chan, Michael J. Meaney

**Affiliations:** 1 Translational Neuroscience, Singapore Institute for Clinical Sciences, Agency for Science, Technology and Research, Singapore, Republic of Singapore; 2 Biostatistics, Singapore Institute for Clinical Sciences, Agency for Science, Technology and Research, Singapore, Republic of Singapore; 3 Yong Loo Lin School of Medicine, National University of Singapore, Singapore, Republic of Singapore; 4 Department of Psychological Medicine, KK Women’s and Children’s Hospital, Singapore, Republic of Singapore; 5 Duke-NUS Medical School, National University of Singapore, Singapore, Republic of Singapore; 6 Department of Educational and Counselling Psychology, McGill University, Montreal, Quebec, Canada; 7 Department of Pediatrics, McGill University, Montreal, Quebec, Canada; 8 Research Institute, McGill University Health Centre, Montreal, Quebec, Canada; 9 Department of Psychiatry, McGill University, Montreal, Quebec, Canada; 10 School of Public Health, University of Montreal, Montreal, Quebec, Canada; 11 Yale Child Study Center and Department of Obstetrics Gynecology and Reproductive Science, Yale School of Medicine, New Haven, Connecticut; 12 Child and Brain Development Program, Canadian Institute for Advanced Research, Ontario, Canada; 13 Owerko Centre for Children’s Neurodevelopment and Mental Health, Departments of Pediatrics, Psychiatry and Community Health Sciences, Alberta Children’s Hospital Research Institute, Cumming School of Medicine, University of Calgary, Calgary, Alberta, Canada; 14 Departments of Pediatrics, Psychiatry and Community Health Sciences, Faculty of Nursing, and Cumming School of Medicine, University of Calgary, Calgary, Alberta, Canada; 15 Department of Obstetrics and Gynecology, National University Hospital, Singapore, Republic of Singapore; 16 Department of Psychiatry, Douglas Mental Health University Institute, McGill University, Montreal, Quebec, Canada

## Abstract

**Importance:**

Depressive symptoms during pregnancy influence the development and health of the offspring, underscoring the need for timely intervention. However, the course of depressive symptoms across the perinatal period remains unclear, thus complicating screening and referral guidelines.

**Objective:**

To examine the course and stability of depressive symptoms across the perinatal period in multiple, ethnically diverse independent observational cohorts.

**Design, Setting, and Participants:**

This cohort study included self-reported depressive symptoms at multiple time points from 7 prospective cohorts spanning 3 continents (United Kingdom: Avon Longitudinal Study of Parents and Children from 1991 to 1995; Canada: Maternal Adversity, Vulnerability and Neurodevelopment from 2003 to 2007; Montreal Antenatal Well-being Study from 2019 to 2022; Alberta Pregnancy Outcomes and Nutrition from 2009 to 2014; and Singapore: Growing Up in Singapore Toward Healthy Outcomes from 2009 to 2013; Singapore Preconception Study of Long-Term Maternal and Child Outcomes from 2015 to 2019; and Mapping Antenatal Maternal Stress from 2019 to 2022). Participants were recruited either during preconception or pregnancy and observed into the postnatal period. All data from each cohort were analyzed from July 2022 to April 2023.

**Main Outcomes and Measures:**

Self-reported depressive symptoms from pregnancy to 2 years following childbirth using either the Edinburgh Postnatal Depression Scale or the Center for Epidemiological Studies Depression were analyzed independently within each cohort using item response theory (IRT) techniques. *K*-means clustering was used to identify groups of participants with similar trajectories.

**Results:**

A total of 11 563 pregnant women (mean [SD] age, 29 [5] years; 569 [4.9%] East Asian women; 304 [2.6%] Southeast Asian women; 10 133 [87.6%] White women) self-reported depressive symptoms from pregnancy to 2 years following childbirth. Analytic methods from Item Response Theory identified 3 groups of mothers based on depressive symptoms: low, mild, and high levels in each of the 7 cohorts. Mothers within and across all cohorts had stable trajectories of maternal depressive symptoms from pregnancy onwards. Mothers with clinical levels of depressive symptoms likewise showed stable trajectories from pregnancy into the postnatal period.

**Conclusions and Relevance:**

In this study, trajectories of depressive symptoms remained stable from pregnancy across the perinatal period, a finding that conflicts with a continuing emphasis on postpartum or postnatal onset of depression that persists in some health policy guidelines. Interventions and public health initiatives should focus on reducing depressive symptoms during pregnancy in addition to following birth.

## Key Points

**Question:**

What is the course and stability of maternal depressive symptoms throughout the perinatal period?

**Findings:**

This cohort study analyzed maternal depressive symptoms trajectories of 11 563 pregnant women from 7 cohorts across 3 continents and showed 3 distinct clusters of mothers with stable low, mild, and high symptom levels throughout the perinatal period. This trajectory was apparent among participants with clinical symptom levels.

**Meaning:**

These findings suggest that interventions, guidelines for care, and public health policies aimed at alleviating maternal depressive symptoms should target both pregnancy and the postnatal period.

## Teaser

This cohort study examines the course and stability of depressive symptoms across the perinatal period in multiple cohorts spanning 3 continents.

## Introduction

Maternal mental health is a modifiable risk factor for poor developmental outcomes in offspring.^1^ However, there remains contradictory information about the prepartum vs postpartum onset of depressive symptoms that continues to complicate clear public health policies about the optimal timing for interventions. While *Diagnostic and Statistical Manual of Mental Disorders* (Fifth Edition) refers to peripartum depression, the medical literature and lexicon typically refer to postpartum or postnatal depression, with multiple influential guidelines referencing the condition as one that follows the birth of the child.^2,3,4^ Some reports^5^ suggest an increased incidence of clinical levels of maternal depression after childbirth. However, it is noteworthy that these studies did not include a prospective longitudinal analysis required to directly address the issue of time of onset. In contrast, prospective longitudinal analyses of large, community-based studies suggest that depressive symptom levels are slightly higher during pregnancy and remain highly stable thereafter.^6,7^ Because maternal depression has serious long-term implications on the child,^1^ it is essential to clearly define when depressive symptoms first emerge to inform timely interventions.

We examined the timing of onset and stability of maternal depressive symptoms using data from 11 563 pregnant women from 7 different, prospective longitudinal community-based cross-continental cohorts, spanning 3 decades. Each cohort included depressive symptoms measured at multiple perinatal time points and analyzed independently. We conducted a trajectory analysis to examine the course and stability of maternal depressive symptoms. We first investigated the stability of the trajectories of maternal depressive symptoms during the perinatal period. We also sought to overcome a weakness of community-based studies, in which the inclusion of large numbers of women with low levels of depressive symptoms might bias toward apparent stability. Thus, we examined the trajectories of maternal depressive symptoms in a subgroup of women meeting clinical cutoffs for depression at any point during the perinatal period.

## Methods

Pregnant women were recruited into 7 prospective observational cohorts, including the United Kingdom Avon Longitudinal Study of Parents and Children (ALSPAC), Canada Alberta Pregnancy Outcomes and Nutrition (APrON) Study, Maternal Adversity, Vulnerability and Neurodevelopment (MAVAN) Study, and Montreal Antenatal Well-Being Study (MAWS), and Singapore Growing Up in Singapore Toward Healthy Outcomes (GUSTO), Singapore Preconception Study of Long-Term Maternal and Child Outcomes (S-PRESTO), and Mapping Antenatal Maternal Stress (MAMS). Written consent was provided by all participants and ethical approval was obtained from the institutional review board in each study (eMethods in Supplement 1).

We harmonized self-reported maternal depressive symptoms data from 11 563 women across these 7 cohorts, from pregnancy up to 2 years postdelivery using either the Edinburgh Postnatal Depression Scale (EPDS)^8^ or the Center for Epidemiological Studies-Depression (CES-D)^9^. Both the EPDS and CES-D are validated antenatal and postnatal screening instruments for maternal depression.^9,10^ The 10-item EPDS (range, 0 to 30) and 20-item CES-D (range, 0 to 60) measure the reported frequency of common depressive symptoms in the past week. The EPDS was administered in all cohorts, except the MAVAN cohort which used CES-D. We also obtained information on maternal age, ethnicity, highest education level attained, and marital status. Details of each cohort study and their respective data collection time points eTables 2 to 7 and the eMethods in Supplement 1.

### Data Analysis

All analyses were performed in R version 4.1.1 (R Project for Statistical Computing).^11^ Missing responses for all cohorts at observed time points were less than 3% and imputed using the R package mice. All individual responses to the EPDS and CES-D from each cohort were analyzed independently at each time point using item response theory (IRT) techniques in the R package eRm. This method uses data optimally as it exploits all individual item responses instead of summarizing in an aggregated score. A latent depression trait estimate was obtained for each participant per time point, hence providing a depressive symptom trajectory for each participant over time. *K*-means clustering was fitted by considering the entire latent trajectory as input for each individual within each cohort to identify groups of participants with similar trajectories. All data from each cohort were analyzed from July 2022 to April 2023.

## Results

We conducted a trajectory analysis of inter-individual differences in depressive symptoms from the 7 prospective cohorts across the perinatal period, including a subgroup of women with probable depression. A total of 11 563 pregnant women (mean [SD] age, 29 [5] years; 569 [4.9%] East Asian women; 304 [2.6%] Southeast Asian women; 10 133 [87.6%] White women) were included. The total number of participants and mean (SD) age of each of the cohorts included: ALSPAC, 8704 participants; mean (SD) age, 29 (5) years; APrON, 953 participants; mean (SD) age, 32 (4) years); MAWS, 710 participants; mean (SD) age, 32 (4) years; GUSTO, 329 participants; mean (SD) age, 31 (5) years; MAVAN, 350 participants; mean (SD) age, 31 (5) years; S-PRESTO, 86 participants; mean (SD) age, 31 (3) years; and MAMS, 431 participants; mean (SD) age, 31 (3) years.

**Figure 1.**
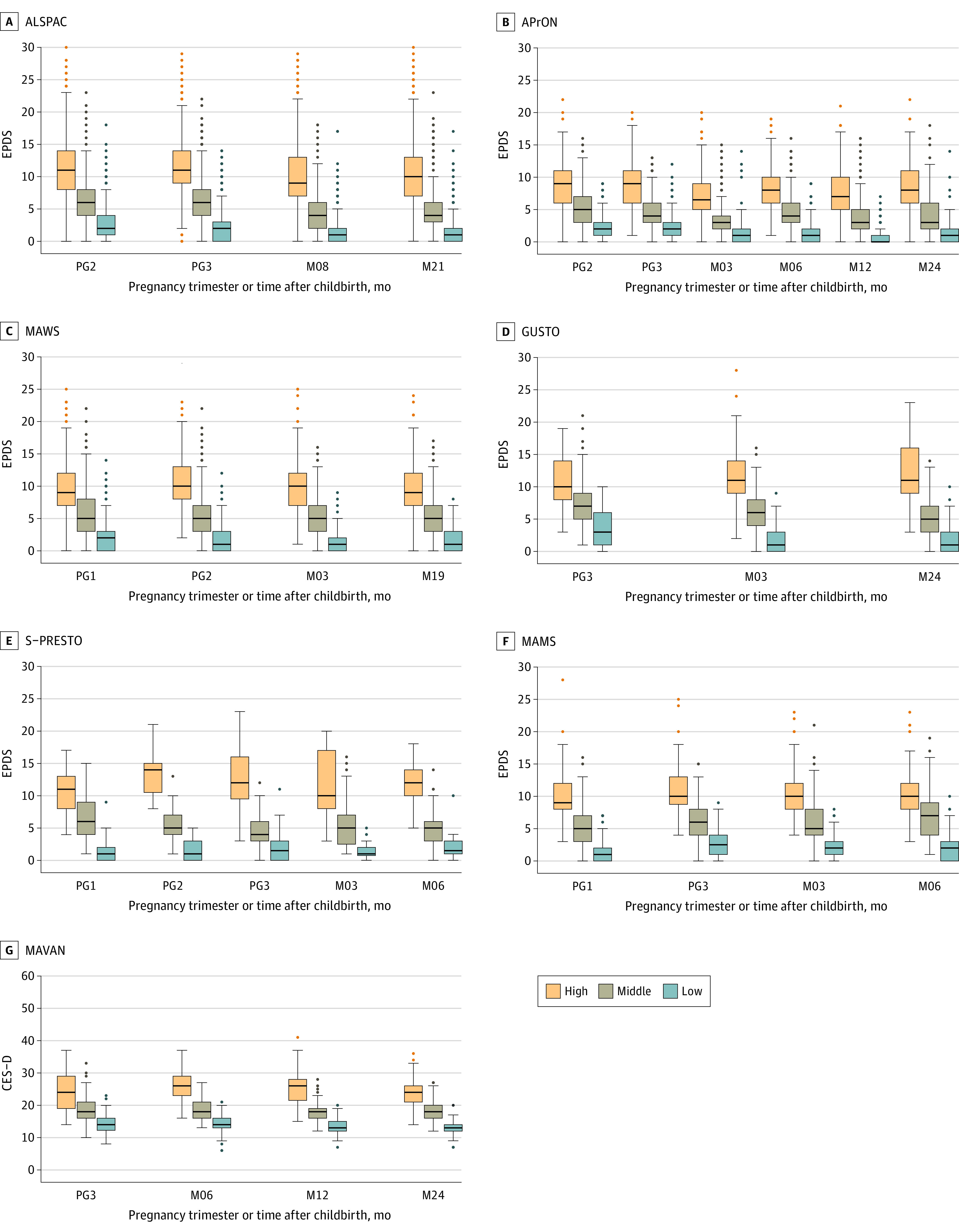
Clusters of Maternal Depressive Symptoms During the Perinatal Period The boxes indicate IQRs; horizontal lines, median; whiskers, 95% CI; and dots, outliers. CES-D, Center for Epidemiological Studies-Depression; EPDS, Edinburgh Postnatal Depression Scale; M03, 3 months after childbirth; M06, 6 months after childbirth; M08, 8 months after childbirth; M12, 12 months after childbirth; M19, 19 months after childbirth; M21, 21 months after childbirth; M24, 24 months after childbirth; PG1, first trimester; PG2, second trimester; PG3, third trimester.

**Figure 2.**
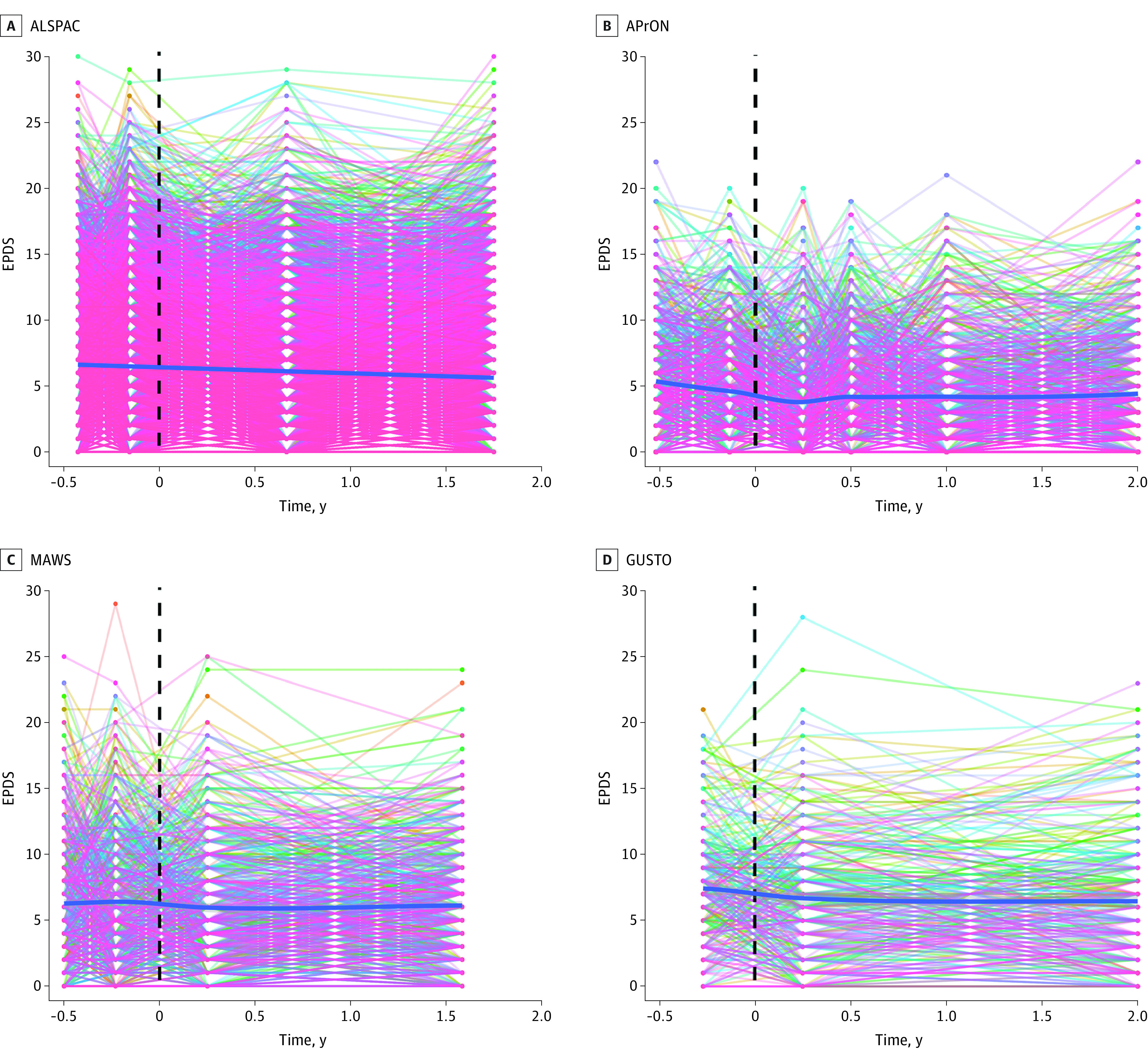
Trajectories of Maternal Depressive Symptoms During the Perinatal Period X-axis refers to time in years from pregnancy to postnatal period. Dashed vertical lines refer to childbirth. Each colored line refers to an individual participant in the cohort. ALSPAC indicates Avon Longitudinal Study of Parents and Children; APrON, Alberta Pregnancy Outcomes and Nutrition; EPDS, Edinburgh Postnatal Depression Scale; GUSTO, Growing Up in Singapore Toward Healthy Outcomes; MAWS, Montreal Antenatal Well-Being Study.

**Figure 3.**
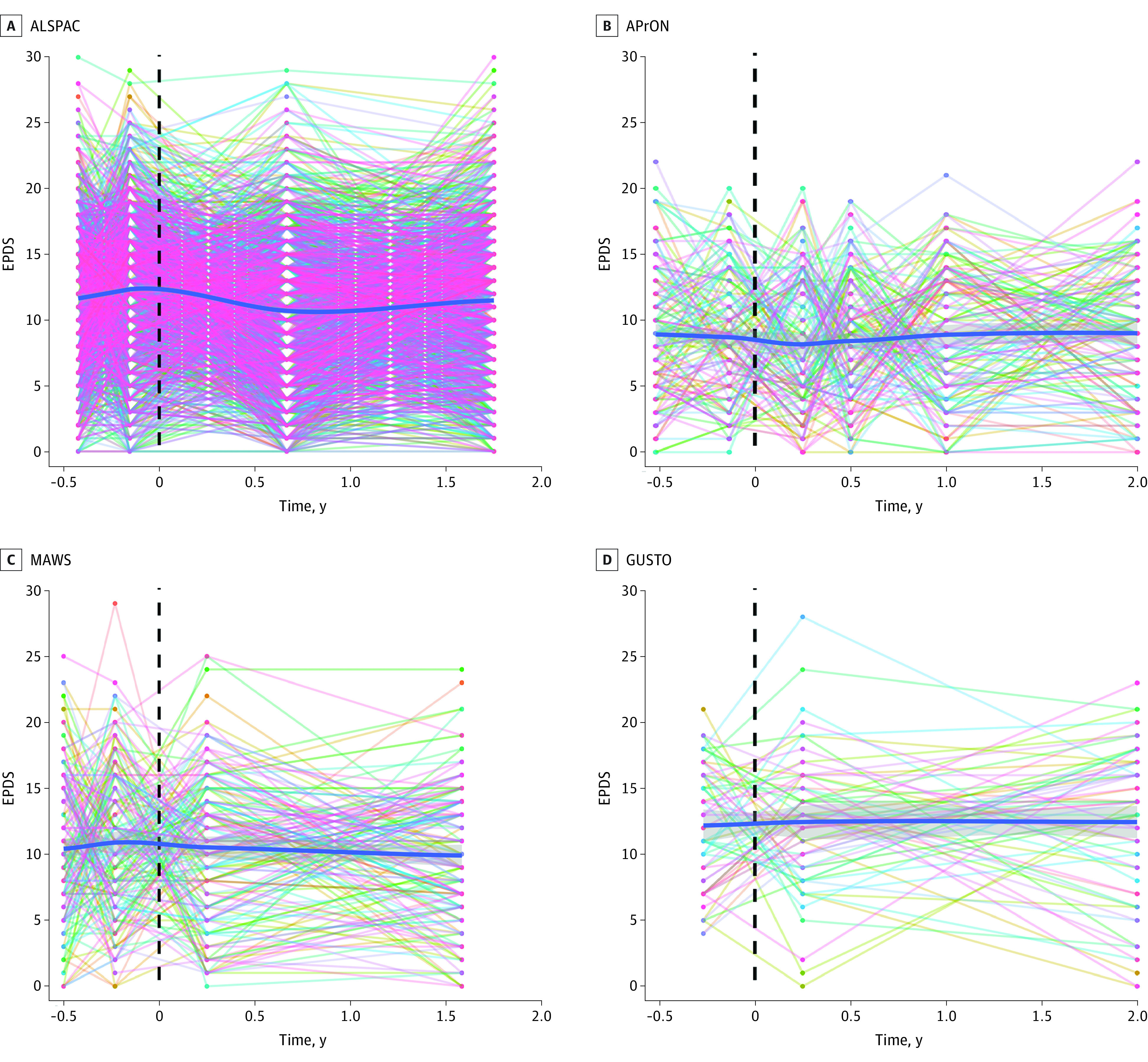
Trajectories of Maternal Depression When Considering Those With Clinically Validated EPDS Cutoffs for Depression Since Pregnancy X-axis refers to time in years from pregnancy to postnatal period. Dashed vertical lines refer to childbirth. Each colored line refers to an individual participant in the cohort. ALSPAC indicates Avon Longitudinal Study of Parents and Children; APrON, Alberta Pregnancy Outcomes and Nutrition; EPDS, Edinburgh Postnatal Depression Scale; GUSTO, Growing Up in Singapore Toward Healthy Outcomes; MAWS, Montreal Antenatal Well-being Study.

Most of the participants in each cohort were either married or had a partner at recruitment: ALSPAC, 6944 women (80.7%); AProN, 928 women (97.4%); MAWS, 667 women (97.2%); GUSTO, 311 women (96.9%); MAVAN, 332 women (94.9%); S-PRESTO, 84 women (100%); MAMS, 420 women (97.4%) The Table contains additional within-cohorts details. *K*-means clustering of the reported depressive symptoms from pregnancy up to 2 years after childbirth revealed 3 consistent trajectory groups of maternal depressive symptoms (low, mild, and high symptom levels) for each of the 7 cohorts (eFigures 1 and 2 in Supplement 1 and Figure 1). Using IRT analyses, the mean trajectory across all individuals in each cohort, including cases passing clinically validated cutoffs for probable depression, remained stable throughout pregnancy up to 2 years after childbirth (Figure 2 and eFigure 3 in Supplement 1). We then examined the perinatal trajectories of maternal depressive symptoms in a subgroup of participants with probable depression at any time point defined by an EPDS score cut-off of 15 or more during pregnancy and/or 13 or more after childbirth.^12^ This inclusion allowed us to examine the maternal depressive symptoms trajectories of women with probable depression either only during pregnancy, only during postnatal or both. This subgroup of mothers also displayed a stable trajectory of maternal depressive symptoms over the perinatal period (Figure 3; eFigure 4 in Supplement 1).

**Table.** Study Characteristics for Data Used From All Cohorts

Characteristics	Participant, No. (%)						
	ALSPAC (n = 8704)	APrON (n = 953)	MAWS (n = 710)	GUSTO (n = 329)	S-PRESTO (n = 86)	MAMS (n = 431)	MAVAN (n = 350)
Maternal age, mean (SD) y	29 (5)	32 (4)	32 (4)	31 (5)	31 (3)	31 (3)	31 (5)
Missing, No.	0	0	0	1	4	0	0
Married or have a partner^a^							
Yes	6944 (80.7)	928 (97.4)	667 (97.2)	311 (96.9)	84 (100.0)	420 (97.4)	332 (94.9)
No	1666 (19.3)	25 (2.6)	12 (1.7)	10 (3.1)	0	11 (2.6)	18 (5.1)
Prefer not to answer	0	0	7 (1.0)	0	0	0	0
Child’s gestational age at birth, mean (SD) wk	39.58 (1.62)	39.32 (1.44)	38.91 (2.00)	38.82 (1.35)	39.09 (0.99)	38.93 (1.40)	39.16 (1.15)
Ethnicity^a^							
African ancestry	68 (0.8)	5 (0.5)	41 (5.8)	0	0	0	10 (3.4)
Caucasian	8481 (98.2)	840 (88.1)	550 (77.5)	0	0	2 (0.5)	260 (89.3)
East Asian	14 (0.2)	42 (4.4)	29 (4.1)	194 (59.0)	69 (80.2)	221 (53.6)	0
Latino	0	14 (1.5)	25 (3.5)	0	0	0	0
Mixed^b^	0	7 (0.7)	3 (0.4)	0	2 (2.3)	1 (0.2)	10 (3.4)
Native or Aboriginal	0	3 (0.3)	4 (0.6)	0	0	0	0
Others^c^	44 (0.5)	4 (0.4)	6 (0.8)	0	0	0	6 (2.1)
South Asian	32 (0.4)	14 (1.5)	18 (2.5)	50 (15.2)	6 (7.0)	30 (7.3)	0
Southeast Asian	0	18 (1.9)	29 (4.1)	85 (25.8)	9 (10.5)	158 (38.3)	5 (1.7)
Did not report	0	6 (0.6)	5 (0.7)	0	0	0	0

Abbreviations: ALSPAC, Avon Longitudinal Study of Parents and Children; APrON, Alberta Pregnancy Outcomes and Nutrition; GUSTO, Growing Up in Singapore Toward Healthy Outcomes; MAMS, Mapping Antenatal Maternal Stress; MAVAN, Maternal Adversity, Vulnerability and Neurodevelopment; MAWS, Montreal Antenatal Well-Being Study; S-PRESTO, Singapore Preconception Study of Long-Term Maternal and Child Outcomes.

^a^ Percentages are rounded off to nearest 0.1%.

^b^ Creole, Malagasy, and any combinations of the other self-reported ethnicities stated.

^c^ Arab, Armenian, Caribbean, Central Asian, Persian, Middle Eastern, and West Asian.

## Discussion

Consistent with previous reports from individual community-based studies, we found 3 stable trajectory groups of women with high, mild, or low levels of depressive symptoms in this study.^12^ Another comparable trajectory analysis reported 4 groups with an added distinction between no and low symptom levels,^7^ which were clustered as a single group in our analysis. Taken together, these findings suggest that maternal depressive symptom levels in community-based cohort studies are apparent during pregnancy and remain stable into the postnatal period. Most importantly, this same pattern of stability across the perinatal period was observed among mothers selected for clinical levels of depressive symptoms at any point over the perinatal period. Thus, the stable trajectories of maternal depressive symptoms across the entire sample are also evident among women experiencing probable depression. The results point to the early antenatal period as a time point for the identification of stable trajectories of maternal depressive symptoms. Public health policies should emphasize the early antenatal period as the optimal timing for interventions targeting maternal depressive symptoms. Finally, our findings underscore the American Psychiatric Association’s recent approach in renaming postpartum depression as peripartum depression.^13^

The strengths of this study are the combination of a detailed statistical analysis, a large sample size from 7 cross-continental cohorts, and repeated measurements of self-reported maternal depressive symptoms. The population-based nature of this study enhances the generalizability of the findings. Additionally, our data were based on prospective maternal self-reports of depressive symptoms eliminating potential bias from retrospective reports. While most of our study participants were White women, the same trajectories are also found in the ethnically diverse Singapore cohorts.

### Limitations

This study has limitations common to community-based cohorts, including the exclusion of individuals using psychotropic medications in some cohorts. Likewise, our study did not include cohorts from the global south. Hence, the findings must be applied with caution to the more vulnerable populations represented in low- and middle-income countries.

## Conclusions

Our findings extend the literature exploring the stability of maternal depressive symptoms and demonstrate that inter-individual differences in maternal depressive symptom levels appear from early pregnancy and remain stable up to 2 years after childbirth. These findings suggest that studies focusing uniquely on postpartum depression miss the optimal timing for examining determinants and intervention benefits for both mother and child. Indeed, recent analyses suggest that maternal depressive symptoms may often predate conception,^14^ highlighting a potentially critical further topic for public health policy revisions.

## Notes

Supplement 1.
eMethods.
**eTable 1. **Overview of Time Points When Maternal Depressive Symptoms Data Were Collected for This Study **eTable 2. **Study Characteristics for Data Used From the ALSPAC Cohort **eTable 3.** Study Characteristics for Data Used From the APrON Cohort **eTable 4.** Study Characteristics for Data Used From the MAWS Cohort **eTable 5.** Study Characteristics for Data Used From the GUSTO Cohort **eTable 6.** Study Characteristics for Data Used From the S-PRESTO Cohort **eTable 7. **Study Characteristics for Data Used From the MAMS Cohort **eTable 8. **Study Characteristics for Data Used From the MAVAN Cohort
eReferences
**eFigure 1.** Scree Plots Showing Total Within-Clusters Sum of Squares (WSS) for All Cohorts Used in *k*-Means Algorithm **eFigure 2. **Scree Plots Showing Total WSS Differences (diff-WSS) Between Consecutive Values of the Number of Clusters **eFigure 3. **Trajectories of Maternal Depressive Symptoms During the Perinatal Period **eFigure 4. **Trajectories of Maternal Depression When Considering Those With Clinically Validated EPDS Cutoffs for Depression Since Pregnancy

Supplement 2.
Data Sharing Statement
