# Supplementary material for: Perinatal Trajectories of Maternal Depressive Symptoms in Prospective, Community-Based Cohorts Across 3 Continents
Source: JAMA Netw Open. 2023 Oct 26;6(10):e2339942. doi: 10.1001/jamanetworkopen.2023.39942 (PMC10603499; doi:10.1001/jamanetworkopen.2023.39942)
Supplement: Supplement 1. — eMethods. eTable 1. Overview of Time Points When Maternal Depressive Symptoms Data Were Collected for This Study eTable 2. Study Characteristics for Data Used From the ALSPAC Cohort eTable 3. Study Characteristics for Data Used From the APrON Cohort eTable 4. Study Characteristics for Data Used From the MAWS Cohort eTable 5. Study Characteristics for Data Used From the GUSTO Cohort eTable 6. Study Characteristics for Data Used From the S-PRESTO Cohort eTable 7. Study Characteristics for Data Used From the MAMS Cohort eTable 8. Study Characteristics for Data Used From the MAVAN Cohort eReferences eFigure 1. Scree Plots Showing Total Within-Clusters Sum of Squares (WSS) for All Cohorts Used in k-Means Algorithm eFigure 2. Scree Plots Showing Total WSS Differences (diff-WSS) Between Consecutive Values of the Number of Clusters eFigure 3. Trajectories of Maternal Depressive Symptoms During the Perinatal Period eFigure 4. Trajectories of Maternal Depression When Considering Those With Clinically Validated EPDS Cutoffs for Depression Since Pregnancy [file jamanetwopen-e2339942-s001.pdf]

Supplemental Online Content

Kee MZL, Cremacshi A, De Iorio M, et al. Perinatal trajectories of maternal depressive symptoms in prospective, community-based cohorts across 3 continents. *JAMA Netw Open*. 2023;6(10):e2339942. doi:10.1001/jamanetworkopen.2023.39942

eMethods.
eFigure 1. Scree Plots Showing Total Within-Clusters Sum of Squares (WSS) for All Cohorts Used in *k*-Means Algorithm
eFigure 2. Scree Plots Showing Total Wss Differences (diff-WSS) Between Consecutive Values of the Number of Clusters
eFigure 3. Trajectories of Maternal Depressive Symptoms During the Perinatal Period
eFigure 4. Trajectories of Maternal Depression When Considering Those With Clinically Validated EPDS Cut-Offs for Depression Since Pregnancy
eTable 1. Overview of Time Points When Maternal Depressive Symptoms Data Were Collected for This Study
eTable 2. Study Characteristics for Data Used From the ALSPAC Cohort
eTable 3. Study Characteristics for Data Used From the APrON Cohort
eTable 4. Study Characteristics for Data Used From the MAWS Cohort
eTable 5. Study Characteristics for Data Used From the GUSTO Cohort
eTable 6. Study Characteristics for Data Used From the S-PRESTO Cohort
eTable 7. Study Characteristics for Data Used From the MAMS Cohort
eTable 8. Study Characteristics for Data Used From the MAVAN Cohort
eReferences

This supplemental material has been provided by the authors to give readers additional information about their work.

© 2023 Kee MZL et al. *JAMA Network Open.*

eMETHODS

Statistical analysis

Scree plots (eFigure 1 and eFigure 2) show the total within-clusters sum of squares (WSS) as a function of the number of clusters used in the *k*-means algorithm. WSS values are obtained by summing the squared deviations of each data point from the cluster center it is assigned to. WSS is an inverse measure of the compactness of a cluster, as points closer to the center yield lower WSS values. Total WSS values are then computed by summing over the different clusters. Total WSS decreases with the number of clusters, as more centers yield partitions with more compact clusters.

Study cohorts' descriptions

Avon Longitudinal Study of Parents and Children (ALSPAC)

The Avon Longitudinal Study of Parents and Children (ALSPAC) cohort is a population-based cohort from Avon county in the United Kingdom.[1–3] Pregnant women in the ALSPAC cohort were recruited if they were Avon residents while pregnant, and their expected delivery dates lie between 1st April 1991 and 31st December 1992 (N = 14,541). Ethical approval for this study was obtained from the ALSPAC Ethics and Law Committee and the Local Research Ethics Committees (a full list of the ethics committees that approved different aspects of the ALSPAC studies is available at http://www.bristol.ac.uk/alspac/researchers/research-ethics/). Data were collected during clinic visits or with postal questionnaires. Informed consent for the use of data collected via questionnaires and clinics was obtained from participants following the recommendations of the ALSPAC Ethics and Law Committee at the time. Please note that the study website contains details of all the data that is available through a fully searchable data dictionary and variable search tool (http://www.bristol.ac.uk/alspac/researchers/our-data/.)

Alberta Pregnancy Outcomes and Nutrition (AProN) Study

The Alberta Pregnancy Outcomes and Nutrition (APrON) Study is a longitudinal cohort of pregnant women and their children living in the central and southern regions of Alberta, Canada.[4] Pregnant women were recruited into the APrON cohort if they were at least 16 years old, able to speak and read English, of less than 27 gestational weeks, and willing to come for on-site clinic visits at the University of Calgary in Calgary, or the University of Alberta, in Edmonton, Alberta. A total of 2189 pregnant women were enrolled into the study between May 2009 and June 2012. 1,945 participants (~88.9%) remained in the study when their children turned three. The APrON study was approved by the University of Calgary Health Research Ethics Board (REB14-1702) and the University of Alberta Health Research Ethics Biomedical Panel (Pro00002954).

Maternal Adversity, Vulnerability and Neurodevelopment (MAVAN)

The Maternal Adversity, Vulnerability and Neurodevelopment (MAVAN) study is a prospective community-based cohort of Canadian mother-child dyads.[5] Pregnant women from Montreal, Quebec, and Hamilton, Ontario were recruited, if they were at least 18 years old and able to speak and read in either English or French. Subjects were excluded from the study if they had serious obstetric complications during pregnancy or delivery of the child, or if the child had extremely low birth weight, was premature (≤ 37 weeks' gestation) or had any congenital disease. Ethical approval for this study was obtained from the Douglas Mental Health University Institute (Montreal) and St-Joseph's Hospital (Hamilton).

Montreal Antenatal Well-being Study (MAWS)

The Montreal Antenatal Well-Being Study (MAWS) is a Quebec-based prospective longitudinal cohort designed to identify the biological, psychological, and social factors that contribute to maternal mental health and family well-being from pregnancy through early childhood. A community-based sample of 1130 pregnant participants between the ages of 18-45 was recruited from August 2019 to March 2021, either in person via phone recruitment from obstetric clinics or self-selection from online advertisements. All mothers have now delivered their babies with a reported 54 losses during pregnancy, mainly due to miscarriages or pregnancy termination. Detailed measures of maternal mental health, psychosocial factors and pregnancy health are collected twice during pregnancy and three times postpartum. Partners of participants were recruited at approximately 18 months postpartum concurrent with a detailed assessment of child development. Ethical approval for this study was obtained from the Research Ethics Board of the CIUSSS de l'Ouest de l'Île de Montréal.[6]

© 2023 Kee MZL et al. *JAMA Network Open.*

Growing Up in Singapore Towards healthy Outcomes (GUSTO)

Pregnant women aged ≥ 18 years were approached to be enrolled into the Growing Up in Singapore Towards healthy Outcomes (GUSTO) longitudinal cohort study from hospital maternity units in Singapore, specifically KK Women's and Children's Hospital (KKH) and National University Hospital (NUH) between June 2009 and October 2010.[7] Pregnant women who were Singapore Citizens or Singapore Permanent Residents, of < 14 gestational weeks, had both the intention to deliver at either hospital and reside in Singapore for the next 5 years were recruited into the study. Additionally, both parents and grandparents of the fetus must be of the same ethnicity. Women who had significant medical conditions (e.g.: Type 1 diabetes mellitus, psychosis), were on certain medication (e.g.: psychotropic drugs), and were on chemotherapy were excluded from the study. 1,450 pregnant women were recruited, leading to 1,209 deliveries. Written informed consent was obtained from all participants. The study was approved by the National Health Care Group Domain Specific Review Board (D/09/02) and SingHealth Centralized Institutional Review Board (2009/280/D).

Singapore PREconception Study of Long-Term Maternal and Child Outcomes (S-PRESTO)

Healthy women aged between 18 to 45 years were recruited into the Singapore PREconception Study of Long-Term Maternal and Child Outcomes (S-PRESTO), a preconception, longitudinal cohort study in Singapore.[8] Recruitment began between February 2015 and October 2017, from both the KK Women's and Children's Hospital and the public. Women of either Chinese, Malay, Indian or any combination of these 3 ethnic groups, who planned to conceive within 1 year of recruitment and had the intention to reside in Singapore for the next 5 years were recruited in the study. Women were excluded from this study if they fulfill any of the following criteria: were actively trying to conceive for more than 18 months; were using oral or implanted contraception; had an *in situ* intrauterine contraceptive device (IUCD) for the past 1 month; were undergoing fertility treatment (apart from those taking clomiphene or letrozole alone in the past 1 month); or had pre-existing health conditions (including established type 1 or type 2 diabetes, on systemic steroids, anticonvulsants, HIV or Hepatitis B or C medication in the past 1 month). 1039 women were recruited, of which 475 became pregnant and 373 women delivered. Written informed consent was provided by participants. Ethical approval was obtained from the SingHealth Centralised Institutional Review Board (reference 2014/692/D). This study has been registered at ClinicalTrials.gov (NCT 03531658).

Mapping Antenatal Maternal Stress (MAMS)

The Mapping Antenatal Maternal Stress (MAMS) is a prospective longitudinal cohort study of pregnant women and their offspring in Singapore. The study recruited pregnant mothers, aged 21 – 40 years, of 14 – 24 gestational weeks, able to speak and read in English, having singletons and who had their obstetrician visits at the National University Hospital in Singapore. Women were excluded from the study if they met any of the following conditions: had pre-existing or a history of psychotic depression, schizophrenia, and bipolar disorders; had a history of thyroid disease; currently on steroids or thyroids medications; became pregnant via any assisted reproduction; or currently enrolled into any interventional randomized controlled trials. The study started in late September 2019 to December 2022. As of July 2022, there are 1329 pregnant women recruited into the study, with 1069 of them having delivered their children. Written informed consent was provided by the participants. The study was approved by the National Healthcare Group Domain Specific Review Board (2018/00967).

© 2023 Kee MZL et al. *JAMA Network Open.*

eFIGURE LEGENDS

eFigure1. Scree plots showing total within-clusters sum of squares (WSS) for all cohorts used in *k*-means algorithm. Red dashed lines refer to the optimal number of *K* clusters (x-axis) identified by an "elbow" in the scree plot, corresponding to the point with the greatest change in the clustering configuration as measured by the total WSS. ALSPAC: Avon Longitudinal Study of Parents and Children; APrON: Alberta Pregnancy Outcomes and Nutrition; MAWS: Montreal Antenatal Wellbeing Study; GUSTO: Growing Up in Singapore Towards healthy Outcomes; S-PRESTO: Singapore PREconception Study of Long-Term Maternal and Child Outcomes; MAMS: Mapping Antenatal Maternal Stress; MAVAN: Maternal Adversity, Vulnerability and Neurodevelopment; EPDS: Edinburgh Postnatal Depression Scale; CES-D: Center for Epidemiological Studies-Depression

eFigure 2. Scree plots showing total WSS differences (diff-WSS) between consecutive values of the number of clusters. These plots approximate the slope of the total WSS, shown in the scree plots of eFigure 1, as a function of the number of clusters. The optimal number of clusters corresponds to the point where the slope of the scree plot stops increasing noticeably. The differences in total WSS are shown in the y-axis, while the x-axis indicates which clustering configurations are considered (i.e., lag *K* refers to the difference between the total WSS obtained with *K+1* and *K* clusters). ALSPAC: Avon Longitudinal Study of Parents and Children; APrON: Alberta Pregnancy Outcomes and Nutrition; MAWS: Montreal Antenatal Wellbeing Study; GUSTO: Growing Up in Singapore Towards healthy Outcomes; S-PRESTO: Singapore PREconception Study of Long-Term Maternal and Child Outcomes; MAMS: Mapping Antenatal Maternal Stress; MAVAN: Maternal Adversity, Vulnerability and Neurodevelopment; EPDS: Edinburgh Postnatal Depression Scale; CES-D: Center for Epidemiological Studies-Depression

eFigure 3. Trajectories of maternal depressive symptoms during the perinatal period. X-axis refers to time in years from pregnancy to postnatal period. Dashed vertical lines refer to childbirth. S-PRESTO: Singapore PREconception Study of Long-Term Maternal and Child Outcomes; MAMS: Mapping Antenatal Maternal Stress; MAVAN: Maternal Adversity, Vulnerability and Neurodevelopment; EPDS: Edinburgh Postnatal Depression Scale; CES-D: Center for Epidemiological Studies Depression

eFigure 4. Trajectories of maternal depression when considering those with clinically-validated EPDS cut-offs for depression since pregnancy. X-axis refers to time in years from pregnancy to postnatal period. Dashed vertical lines refer to childbirth. S-PRESTO: Singapore PREconception Study of Long-Term Maternal and Child Outcomes; MAMS: Mapping Antenatal Maternal Stress; EPDS: Edinburgh Postnatal Depression Scale.

© 2023 Kee MZL et al. *JAMA Network Open.*

eTable 1. Overview of time points when maternal depressive symptoms data were collected for this study

Cohort	Measure	Timepoints collected
ALSPAC	EPDS	PG2, PG3, M08, M21
APrON	EPDS	PG2, PG4, M03, M06, M12, M24
MAWS	EPDS	PG1, PG2, PG3, M03, M19
GUSTO	EPDS	PG3, M03, M24
S-PRESTO	EPDS	PG1, PG2, PG3, M03, M06
MAMS	EPDS	PG1, PG3, M03, M06
MAVAN	CES-D	PG3, M06, M12, M24

ALSPAC: Avon Longitudinal Study of Parents and Children; APrON: Alberta Pregnancy Outcomes and Nutrition (APrON); MAVAN: Maternal Adversity, Vulnerability and Neurodevelopment; MAWS: Montreal Antenatal Wellbeing Study; GUSTO: Growing Up in Singapore Towards healthy Outcomes; S-PRESTO: Singapore PREconception Study of Long-Term Maternal and Child Outcomes; MAMS: Mapping Antenatal Maternal Stress; EPDS: Edinburgh Postnatal Depression Scale; CES-D: Center for Epidemiological Studies-Depression. PG1, PG2 and PG3 represent 1st, 2nd and 3rd trimester of pregnancy respectively. M03, M06, M08, M12, M19, M21 and M24 represent 3-, 6-, 8-, 12-, 19-, 21- and 24-months postpartum respectively.

© 2023 Kee MZL et al. *JAMA Network Open.*

eTable 2. Study characteristics for data used from the ALSPAC cohort

	**Common IDs** (N=8704)	**PG2** (N=12283)	**PG3** (N=12160)	**M08** (N=11268)	**M21** (N=10326)
**Marital status,** N (%)*					
Missing	94	461	331	317	256
Married	6944 (80.7%)	9036 (76.4%)	9071 (76.7%)	8540 (78.0%)	7926 (78.7%)
Not married	1666 (19.3%)	2786 (23.6%)	2758 (23.3%)	2411 (22.0%)	2144 (21.3%)
**Maternal age (Years),** Means ± SDs	29 ± 5	28 ± 5	28 ± 5	28 ± 5	29 ± 5
Missing	0	97	16	0	0
**Ethnic group,** N (%)*					
Missing	65	1073	139	502	391
African ancestry	68 (0.8%)	114 (1.0%)	126 (1.0%)	102 (0.9%)	84 (0.8%)
Caucasian	8481 (98.2%)	10935 (97.5%)	11713 (97.4%)	10530 (97.8%)	9735 (98.0%)
East Asian	14 (0.2%)	25 (0.2%)	30 (0.2%)	20 (0.2%)	17 (0.2%)
South Asian	32 (0.4%)	66 (0.6%)	77 (0.6%)	52 (0.5%)	47 (0.5%)
Others	44 (0.5%)	**70 (0.6%)**	**75 (0.6%)**	**62 (0.6%)**	**52 (0.5%)**
**Maternal highest education level attained,** N (%)*					
Missing	36	996	71	449	349
CSE and below	1373 (15.8%)	2201 (19.5%)	2411 (19.9%)	1925 (17.8%)	1675 (16.8%)
Vocational	787 (9.1%)	1110 (9.8%)	1196 (9.9%)	1027 (9.5%)	910 (9.1%)
O-levels	3127 (36.1%)	3962 (35.1%)	4204 (34.8%)	3837 (35.5%)	3551 (35.6%)
A-levels	2132 (24.6%)	2558 (22.7%)	2716 (22.5%)	2538 (23.5%)	2417 (24.2%)
Degree	1249 (14.4%)	1456 (12.9%)	1562 (12.9%)	1492 (13.8%)	1424 (14.3%)

*Percentages are rounded off to nearest 0.1%. Common IDs refers to participants found at all stated timepoints and hence, used for analyses. PG2 and PG3 represent 2nd and 3rd trimester of pregnancy respectively. M08 and M21 represent 8- and 21-months postpartum respectively.

© 2023 Kee MZL et al. *JAMA Network Open.*

eTable 3. Study characteristics for data used from the APrON cohort

	**Common IDs** (N=953)	**PG2** (N=2098)	**PG3** (N=1821)	**M03** (N=1517)	**M06** (N=1770)	**M12** (N=1288)	**M24** (N=1278)
**Marital status,** N (%)*							
Married	928 (97.4%)	2009 (95.9%)	1758 (96.5%)	1472 (97.0%)	1714 (96.8%)	1250 (97.0%)	1240 (97.1%)
Not married	25 (2.6%)	85 (4.1%)	63 (3.5%)	45 (3.0%)	56 (3.2%)	38 (3.0%)	37 (2.9%)
**Maternal age (Years),** Means ± SDs	32 ± 4	32 ± 4	32 ± 4	32 ± 4	32 ± 4	32 ± 4	32 ± 4
**Ethnic group,** N (%)*							
African ancestry	5 (0.5%)	32 (1.5%)	22 (1.2%)	16 (1.1%)	21 (1.2%)	10 (0.8%)	12 (0.9%)
Caucasian	840 (88.1%)	1667 (79.5%)	1457 (80.0%)	1245 (82.1%)	1430 (80.8%)	1083 (84.1%)	1096 (85.8%)
East Asian	42 (4.4%)	89 (4.2%)	83 (4.6%)	69 (4.5%)	74 (4.2%)	55 (4.3%)	60 (4.7%)
Latinos	14 (1.5%)	63 (3.0%)	52 (2.9%)	34 (2.2%)	54 (3.1%)	27 (2.1%)	28 (2.2%)
Mixed [a]	7 (0.7%)	18 (0.9%)	15 (0.8%)	14 (0.9%)	15 (0.8%)	11 (0.9%)	8 (0.6%)
Native/Aboriginal	3 (0.3%)	22 (1.0%)	16 (0.9%)	8 (0.5%)	13 (0.7%)	7 (0.5%)	5 (0.4%)
Others [b]	4 (0.4%)	14 (0.7%)	11 (0.6%)	10 (0.7%)	10 (0.6%)	7 (0.5%)	6 (0.5%)
South Asian	14 (1.5%)	32 (1.5%)	27 (1.5%)	21 (1.4%)	26 (1.5%)	19 (1.5%)	16 (1.3%)
Southeast Asian	18 (1.9%)	138 (6.6%)	110 (6.0%)	83 (5.5%)	100 (5.6%)	55 (4.3%)	32 (2.5%)
Did not report	6 (0.6%)	23 (1.1%)	28 (1.5%)	17 (1.1%)	27 (1.5%)	14 (1.1%)	15 (1.2%)
**Maternal highest education level attained,** N (%)*							
Below Degree	222 (23.3%)	659 (31.8%)	519 (28.9%)	413 (27.5%)	506 (28.9%)	333 (26.0%)	316 (24.9%)
Degree	729 (76.7%)	1414 (68.2%)	1279 (71.1%)	1088 (72.5%)	1246 (71.1%)	947 (74.0%)	955 (75.1%)

[a] refers to any combinations of the above-mentioned self-reported ethnicities. [b] refers to Arab, Caribbean, Central Asian and West Asian. *Percentages are rounded off to nearest 0.1%. Common IDs refers to participants found at all stated timepoints and hence, used for analyses. PG2 and PG3 represent 2nd and 3rd trimester of pregnancy respectively. M03, M06, M12 and M24 represent 3-, 6-, 12- and 24-months postpartum respectively.

© 2023 Kee MZL et al. *JAMA Network Open.*

eTable 4. Study characteristics for data used from the MAWS cohort

	**Common IDs** (N=710)	**PG1** (N=1070)	**PG2** (N=932)	**M03** (N=869)	**M19** (N=802)
**Has a partner,** N (%)*					
Yes	667 (97.2%)	993 (96.0%)	858 (96.8%)	806 (97.1%)	742 (97.0%)
No	12 (1.7%)	29 (2.8%)	19 (2.1%)	16 (1.9%)	14 (1.8%)
Prefer not to answer	7 (1.0%)	12 (1.2%)	9 (1.0%)	8 (1.0%)	9 (1.2%)
**Maternal age (Years),** Means ± SDs	32 ± 4	32 ± 4	32 ± 4	32 ± 4	32 ± 4
Missing (N)	0	64	2	20	13
**Ethnic group,** N (%)*					
African ancestry	41 (5.8%)	85 (8.0%)	59 (6.5%)	50 (5.9%)	50 (6.4%)
Caucasian	550 (78.0%)	778 (73.4%)	693 (76.2%)	650 (76.6%)	596 (76.2%)
East Asian	29 (4.1%)	41 (3.9%)	35 (3.9%)	38 (4.5%)	32 (4.1%)
Latinos	25 (3.5%)	43 (4.1%)	33 (3.6%)	29 (3.4%)	27 (3.5%)
Mixed [a]	1 (0.1%)	3 (0.3%)	1 (0.1%)	1 (0.1%)	3 (0.4%)
Native/Aboriginal	4 (0.6%)	8 (0.8%)	7 (0.8%)	5 (0.6%)	5 (0.6%)
Others [b]	8 (1.1%)	12 (1.1%)	10 (1.1%)	9 (1.1%)	9 (1.1%)
South Asian	18 (2.6%)	36 (3.4%)	29 (3.2%)	24 (2.8%)	21 (2.7%)
Southeast Asian	29 (4.1%)	54 (5.1%)	42 (4.6%)	43 (5.1%)	39 (5.0%)
Did not report	5 (0.7%)	10 (0.9%)	8 (0.9%)	8 (0.9%)	8 (1.0%)
**Maternal highest education level attained,** N (%)*					
Secondary 5 (High school, Grade 12 or 13) or lower	45 (6.3%)	87 (8.1%)	67 (7.3%)	58 (6.8%)	53 (6.7%)
Trade certificate/ diploma from vocational school/ apprentice training	55 (7.7%)	96 (9.0%)	72 (7.9%)	65 (7.6%)	64 (8.1%)
Non-university certificate/diploma from community college	79 (11.1%)	121 (11.3%)	104 (11.3%)	98 (11.4%)	87 (11.0%)
University certificate below bachelors' level	38 (5.4%)	59 (5.5%)	52 (5.7%)	47 (5.5%)	46 (5.8%)
Bachelor's degree and above	488 (68.7%)	695 (65.0%)	612 (66.7%)	582 (67.9%)	534 (67.6%)
Prefer not to answer	5 (0.7%)	12 (1.1%)	10 (1.1%)	7 (0.8%)	6 (0.8%)

[a] refers to Creole or any combinations of the above-mentioned self-reported ethnicities. [b] refers to Arab, Armenian, Persian, Malagasy, Middle Eastern, West Asian. *Percentages are rounded off to nearest 0.1%. Common IDs refers to participants found at all stated timepoints and hence, used for analyses. PG2 and PG3 represent 2nd and 3rd trimester of pregnancy respectively. M03 and M19 represent 3- and 19-months postpartum respectively.

© 2023 Kee MZL et al. *JAMA Network Open.*

eTable 5. Study characteristics for data used from the GUSTO cohort

	**Common IDs** (N=329)	**PG3** (N=1190)	**M03** (N=796)	**M24** (N=419)
**Marital status,** N (%)*				
Married	311 (96.9%)	1116 (96.5%)	740 (96.5%)	400 (97.3%)
Not married	10 (3.1%)	41 (3.5%)	27 (3.5%)	11 (2.7%)
**Maternal age (Years),** Means ± SDs	31 ± 5	31 ± 5	31 ± 5	31 ± 5
Missing	1	8	11	1
**Ethnic group,** N (%)*				
East Asian	194 (59.0%)	685 (57.6%)	454 (57.0%)	245 (58.5%)
South Asian	50 (15.2%)	214 (18.0%)	136 (17.1%)	64 (15.3%)
Southeast Asian	85 (25.8%)	291 (24.5%)	206 (25.9%)	110 (26.3%)
**Maternal highest education level attained,** N (%)*				
Pre-Tertiary	114 (46.7%)	407 (50.3%)	274 (49.2%)	149 (49.0%)
University degree and above	130 (53.3%)	402 (49.7%)	283 (50.8%)	155 (51.0%)

*Percentages are rounded off to nearest 0.1%. Common IDs refers to participants found at all stated timepoints and hence, used for analyses. PG3 represent 3rd trimester of pregnancy respectively. M03 and M24 represent 3- and 24-months postpartum respectively.

© 2023 Kee MZL et al. *JAMA Network Open.*

eTable 6. Study characteristics for data used from the S-PRESTO cohort

	**Common IDs** (N=86)	**PG1** (N=275)	**PG2** (N=301)	**PG3** (N=248)	**M03** (N=218)	**M06** (N=183)
**Marital status,** N (%)*						
Married	84 (100.0%)	233 (99.6%)	292 (100.0%)	240 (99.6%)	213 (100.0%)	180 (100.0%)
Not married	0 (0.0%)	1 (0.4%)	0 (0.0%)	1 (0.4%)	0 (0.0%)	0 (0.0%)
**Maternal age (Years),** Means ± SDs	32 ± 3	31 ± 3	31 ± 3	31 ± 3	31 ± 3	31 ± 3
Missing	4	41	7	6	7	5
**Ethnic group,** N (%)*						
East Asian	69 (80.2%)	207 (75.3%)	235 (78.1%)	190 (76.6%)	165 (75.7%)	143 (78.1%)
South Asian	6 (7.0%)	25 (9.1%)	21 (7.0%)	14 (5.6%)	15 (6.9%)	9 (4.9%)
Southeast Asian	9 (10.5%)	35 (12.7%)	38 (12.6%)	36 (14.5%)	33 (15.1%)	27 (14.8%)
Mixed [a]	2 (2.3%)	8 (2.9%)	7 (2.3%)	8 (3.2%)	5 (2.3%)	4 (2.2%)
**Maternal highest education level attained,** N (%)*						
Secondary and below	0 (0.0%)	6 (2.2%)	8 (2.7%)	5 (2.0%)	4 (1.8%)	3 (1.6%)
Pre-Tertiary	13 (15.1%)	64 (23.3%)	75 (24.9%)	62 (25.0%)	49 (22.5%)	41 (22.4%)
University degree and above	73 (84.9%)	205 (74.5%)	218 (72.4%)	181 (73.0%)	165 (75.7%)	139 (76.0%)

[a] refers to any combinations of the above-mentioned self-reported ethnicities. *Percentages are rounded off to nearest 0.1%. Common IDs refers to participants found at all stated timepoints and hence, used for analyses. PG1, PG2 and PG3 represent 1st, 2nd and 3rd trimester of pregnancy respectively. M03 and M6 represent 3- and 6-months postpartum respectively.

© 2023 Kee MZL et al. *JAMA Network Open.*

eTable 7. Study characteristics for data used from the MAMS cohort

	**Common IDs** (N=431)	**PG1** (N=1177)	**PG3** (N=1005)	**M03** (N=615)	**M06** (N=500)
**Marital status,** N (%)*					
Married	420 (97.4%)	1127 (97.6%)	972 (97.2%)	601 (97.7%)	484 (97.0%)
Not married	11 (2.6%)	28 (2.4%)	28 (2.8%)	14 (2.3%)	15 (3.0%)
**Maternal age (Years),** Means ± SDs	31 ± 3	31 ± 4	31 ± 4	31 ± 3	30 ± 3
**Ethnic group,** N (%)*					
Caucasian	2 (0.5%)	6 (0.5%)	4 (0.4%)	4 (0.7%)	2 (0.4%)
East Asian	221 (53.6%)	516 (47.0%)	473 (49.5%)	298 (50.8%)	246 (51.4%)
South Asian	30 (7.3%)	110 (10.0%)	86 (9.0%)	54 (9.2%)	37 (7.7%)
Southeast Asian	158 (38.3%)	465 (42.3%)	390 (40.8%)	230 (39.2%)	192 (40.1%)
Mixed [a]	1 (0.2%)	2 (0.2%)	2 (0.2%)	1 (0.2%)	2 (0.4%)
**Maternal highest education level attained,** N (%)*					
Secondary and below	31 (7.2%)	85 (7.4%)	72 (7.2%)	41 (6.7%)	37 (7.4%)
Pre-Tertiary	112 (26.0%)	332 (28.7%)	274 (27.4%)	167 (27.2%)	135 (27.1%)
University degree and above	288 (66.8%)	738 (63.9%)	654 (65.4%)	407 (66.2%)	327 (65.5%)

[a] refers to any combinations of the above-mentioned self-reported ethnicities. * Percentages are rounded off to nearest 0.1%. Common IDs refers to participants found at all stated timepoints and hence, used for analyses. PG1 and PG3 represent 1st and 3rd trimester of pregnancy respectively. M03 and M6 represent 3- and 6-months postpartum respectively.

© 2023 Kee MZL et al. *JAMA Network Open.*

eTable 8. Study characteristics for data used from the MAVAN cohort

	**Common IDs** (N=350)	**PG3** (N=399)	**M06** (N=478)	**M12** (N=470)	**M24** (N=446)
**Has a partner,** N (%)*					
No	18 (5.1%)	21 (5.3%)	24 (5.0%)	20 (4.9%)	19 (5.0%)
Yes	332 (94.9%)	378 (94.7%)	454 (95.0%)	387 (95.1%)	364 (95.0%)
**Maternal age (Years)**	31 ± 5	31 ± 5	31 ± 5	31 ± 5	31 ± 5
Missing	0	0	0	3	3
**Ethnic group,** N (%)*					
African ancestry	10 (3.4%)	14 (4.3%)	15 (4.5%)	22 (6.0%)	20 (5.4%)
Caucasian	260 (89.3%)	288 (87.8%)	292 (87.7%)	316 (85.6%)	319 (86.2%)
Latinos	0 (0.0%)	0 (0.0%)	0 (0.0%)	0 (0.0%)	0 (0.0%)
Mixed [a]	10 (3.4%)	12 (3.7%)	13 (3.9%)	18 (4.9%)	15 (4.1%)
Others [b]	6 (2.1%)	9 (2.7%)	8 (2.4%)	7 (1.9%)	10 (2.7%)
Southeast Asian	5 (1.7%)	5 (1.5%)	5 (1.5%)	6 (1.6%)	6 (1.6%)
**Maternal highest education level attained,** N (%)*					
High school or below	77 (22.0%)	95 (23.8%)	123 (25.7%)	111 (23.9%)	106 (24.0%)
College	90 (25.7%)	98 (24.6%)	114 (23.8%)	112 (24.1%)	105 (23.8%)
University degree or higher	183 (52.3%)	206 (51.6%)	241 (50.4%)	241 (51.9%)	230 (52.2%)

[a] refers to any combinations of the above-mentioned self-reported ethnicities. [b] refers to Arab. *Percentages are rounded off to nearest 0.1%. Common IDs refers to participants found at all stated timepoints and hence, used for analyses. PG3 represents 3rd trimester of pregnancy respectively. M06, M12 and M24 represent 6-, 12- and 24-months postpartum respectively.

© 2023 Kee MZL et al. *JAMA Network Open.*

eReferences

1. Boyd A, Golding J, Macleod J, et al. Cohort profile: The 'Children of the 90s'-The index offspring of the avon longitudinal study of parents and children. *International Journal of Epidemiology*. 2013;42(1):111-127. doi:10.1093/ije/dys064

2. Fraser A, Macdonald-wallis C, Tilling K, et al. Cohort profile: The avon longitudinal study of parents and children: ALSPAC mothers cohort. *International Journal of Epidemiology*. 2013;42(1):97-110. doi:10.1093/ije/dys066

3. Northstone K, Lewcock M, Groom A, et al. The Avon Longitudinal Study of Parents and Children (ALSPAC): an update on the enrolled sample of index children in 2019 [version 1; peer review: 2 approved]. *Wellcome Open Research*. 2019;4(May):1-10. doi:10.12688/wellcomeopenres.15132.1

4. Kaplan BJ, Giesbrecht GF, Leung BMY, et al. The Alberta Pregnancy Outcomes and Nutrition (APrON) cohort study: Rationale and methods. *Maternal and Child Nutrition*. 2014;10(1):44-60. doi:10.1111/j.1740-8709.2012.00433.x

5. O'Donnell KA, Gaudreau H, Colalillo S, et al. The maternal adversity, vulnerability and neurodevelopment project: Theory and methodology. *Canadian Journal of Psychiatry*. 2014;59(9):497-508. doi:10.1177/070674371405900906

6. Davis K, Robert CH, Montreuil T, et al. *The Montreal Antenatal Well-Being Study (MAWS): A Prospective Longitudinal Study of Perinatal Mental Health.* PsyArXiv; 2023. doi:10.31234/osf.io/jup5a

7. Soh SE, Tint MT, Gluckman PD, et al. Cohort Profile: Growing Up in Singapore Towards healthy Outcomes (GUSTO) birth cohort study. *International Journal of Epidemiology*. 2014;43(5):1401-1409. doi:10.1093/ije/dyt125

8. Loo EXL, Soh SE, Loy SL, et al. Cohort profile: Singapore Preconception Study of Long-Term Maternal and Child Outcomes (S-PRESTO). *European Journal of Epidemiology*. 2021;36(1):129-142. doi:10.1007/s10654-020-00697-2

© 2023 Kee MZL et al. *JAMA Network Open.*

eFigure 1

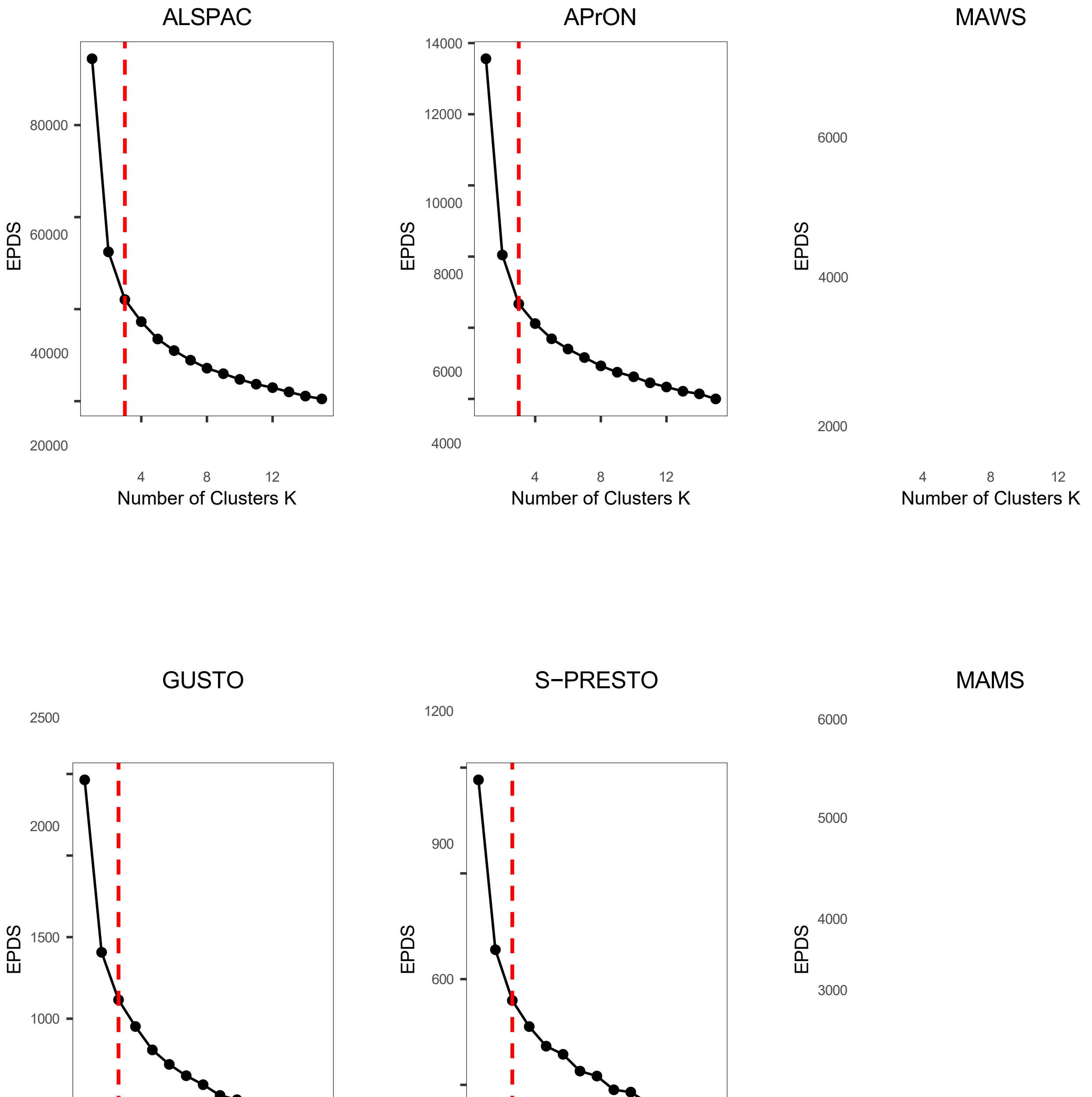

© 2023 Kee MZL et al. *JAMA Network Open.*

eFigure 2

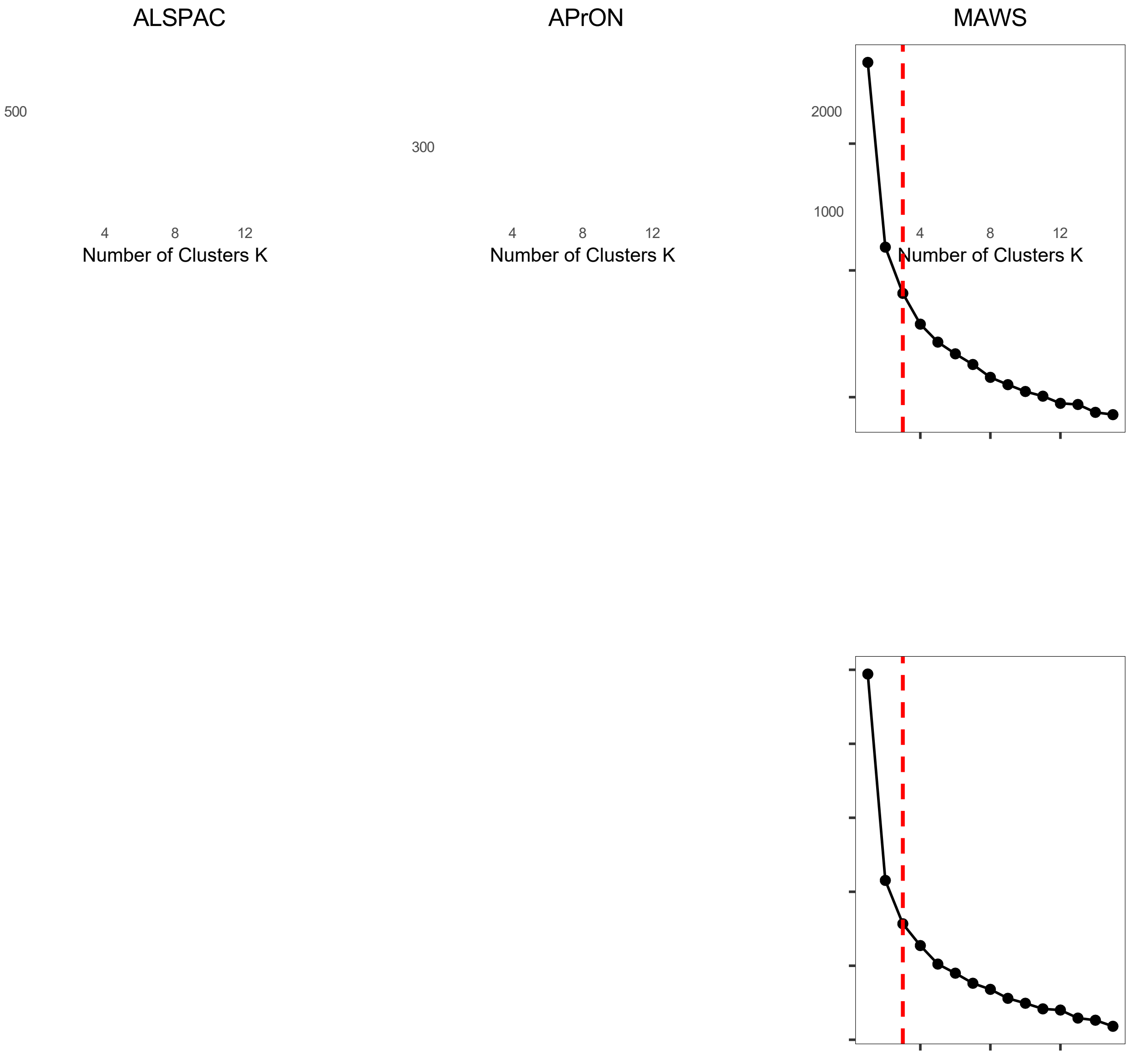

© 2023 Kee MZL et al. *JAMA Network Open.*

eFigure 3

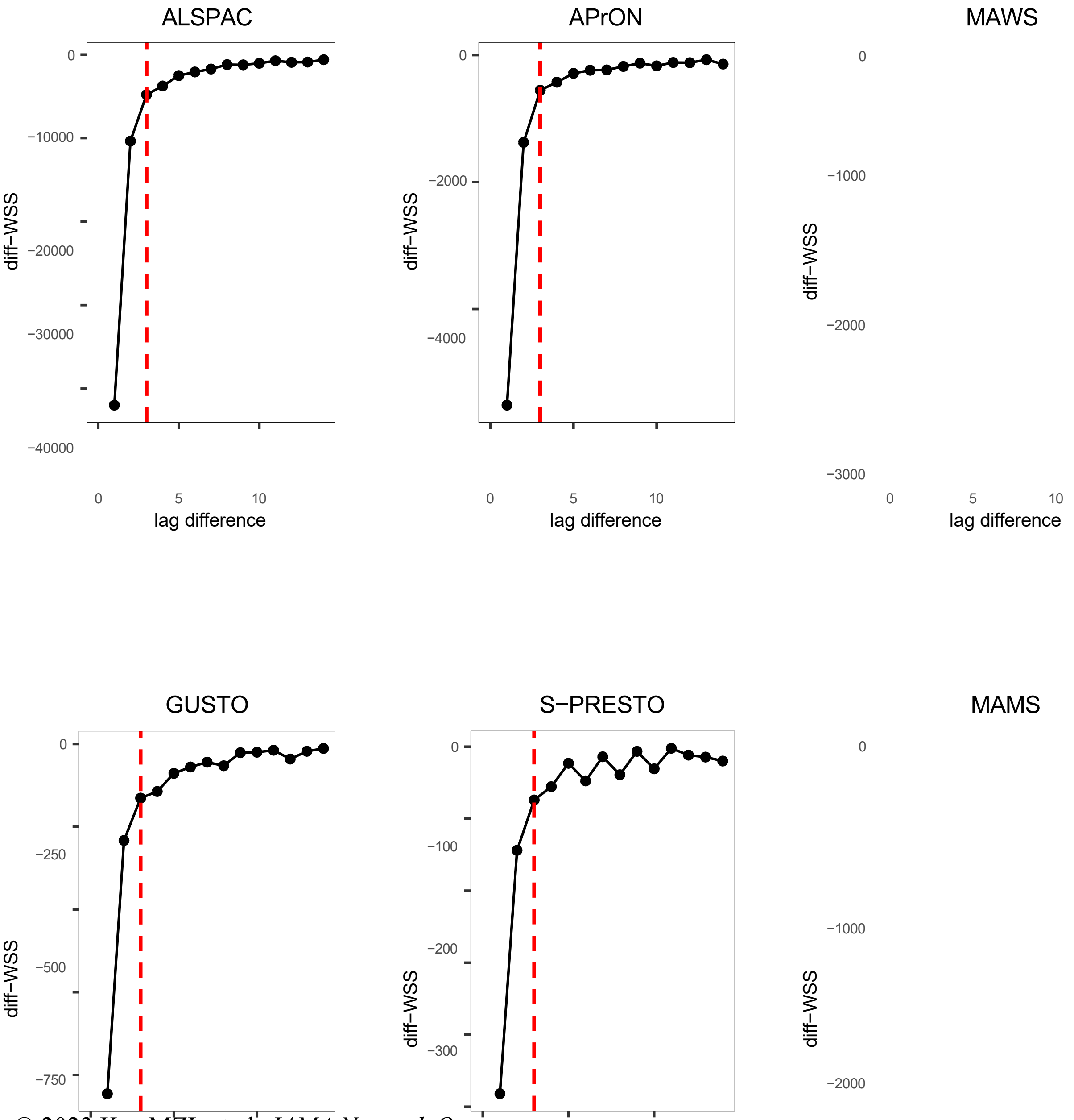

© 2023 Kee MZL et al. *JAMA Network Open.*

eFigure 4

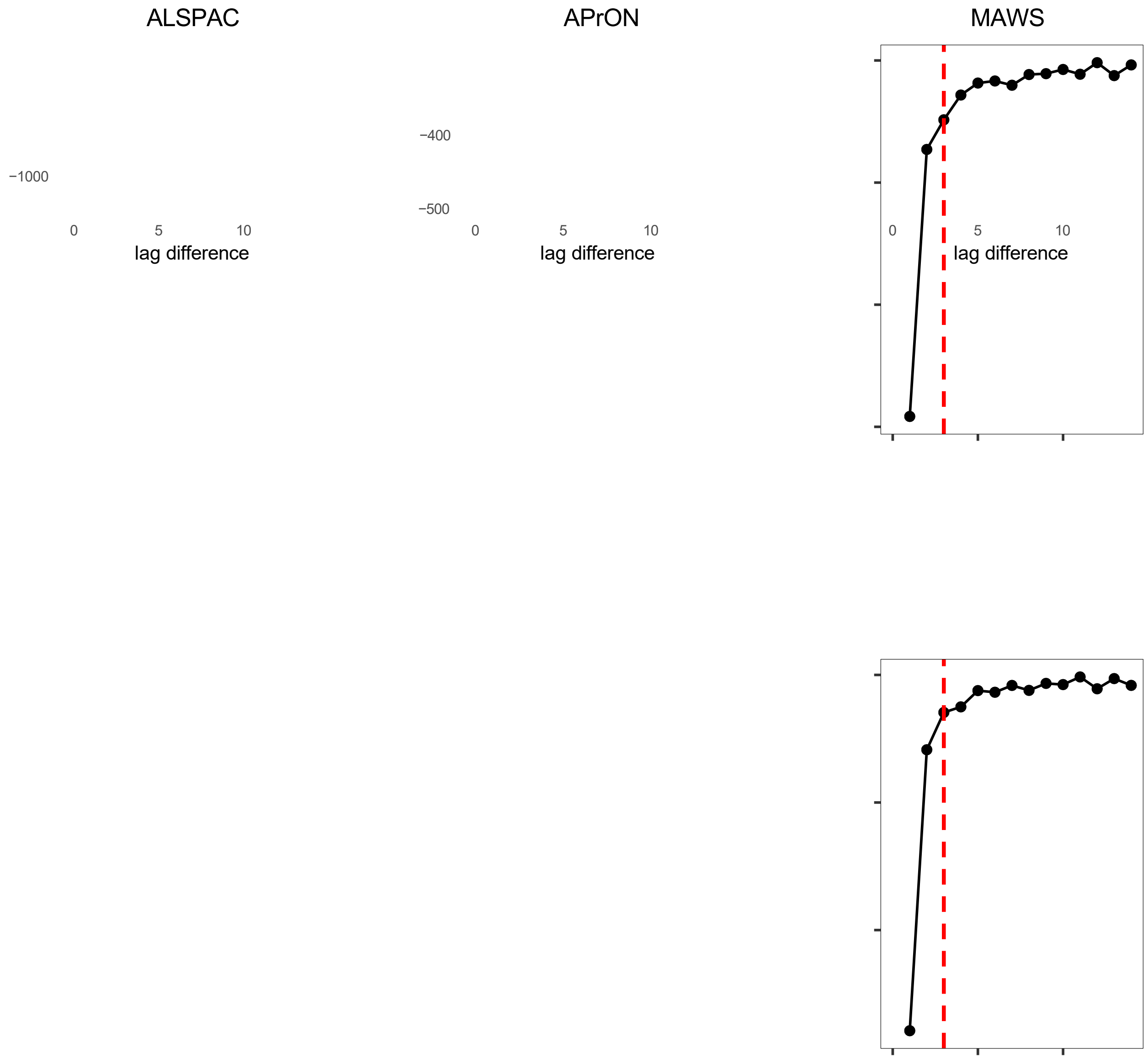

© 2023 Kee MZL et al. *JAMA Network Open.*

eFigure 3.

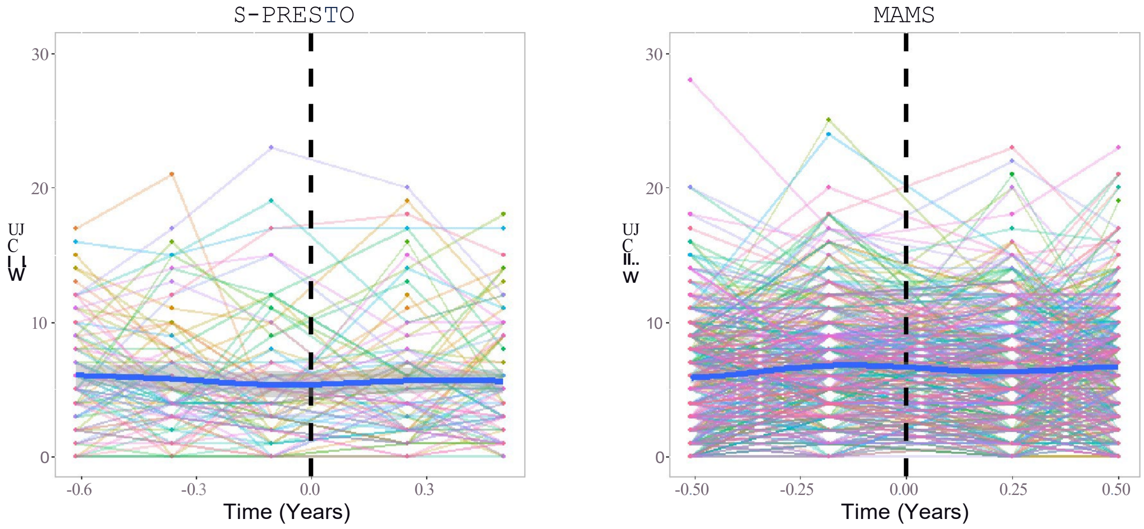

eFigure 4.

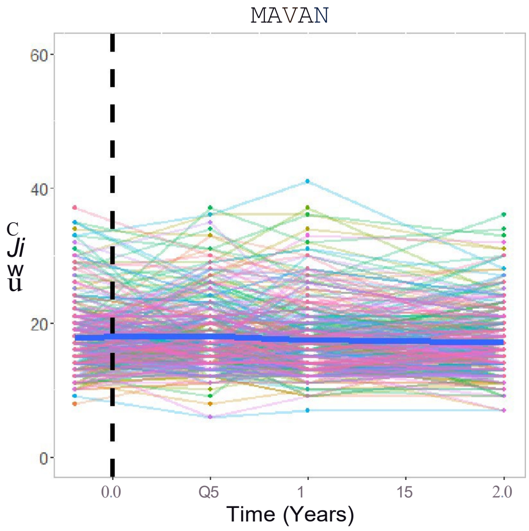

© 2023 Kee MZL et al. *JAMA Network Open.*

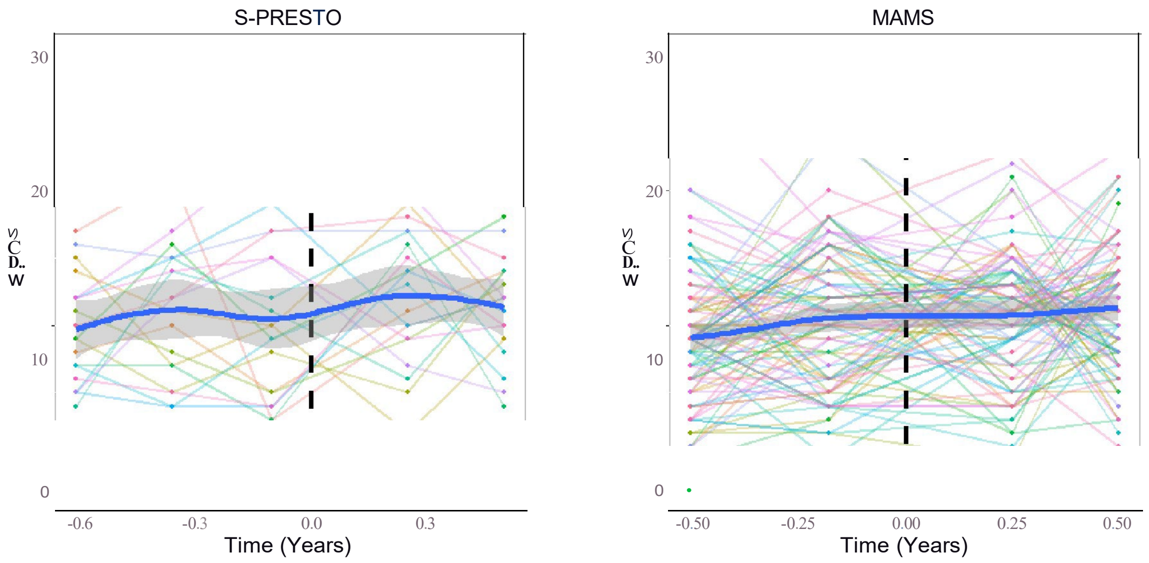

© 2023 Kee MZL et al. *JAMA Network Open.*
