# Supplementary material for: Perinatal Trajectories of Maternal Depressive Symptoms in Prospective, Community-Based Cohorts Across 3 Continents
Source: JAMA Netw Open. 2023 Oct 26;6(10):e2339942. doi: 10.1001/jamanetworkopen.2023.39942 (PMC10603499; doi:10.1001/jamanetworkopen.2023.39942)
Supplement: Supplement 2. — Data Sharing Statement [file jamanetwopen-e2339942-s002.pdf]

Data Sharing Statement

Kee. Perinatal Trajectories of Maternal Depressive Symptoms in Prospective, Community-Based Cohorts Across 3 Continents. *JAMA Netw Open*. Published October 26, 2023. doi:10.1001/jamanetworkopen.2023.39942

Data
Data available: No

Additional Information
Explanation for why data not available: The data that support the findings of this study are available from all respective cohorts but restrictions apply to the availability of these data, which were used under license for the current study, and so are not publicly available. Data are, however, available from the authors upon reasonable request and with permission from the respective cohorts.
